# Analysis of transcribed sequences from young and mature zebrafish thrombocytes

**DOI:** 10.1371/journal.pone.0264776

**Published:** 2022-03-23

**Authors:** Weam Fallatah, Ronika De, David Burks, Rajeev K. Azad, Pudur Jagadeeswaran

**Affiliations:** 1 Department of Biological Sciences, University of North Texas, Denton, TX, United States of America; 2 BioDiscovery Institute, University of North Texas, Denton, TX, United States of America; 3 Department of Mathematics, University of North Texas, Denton, TX, United States of America; Chang Gung University, TAIWAN

## Abstract

The zebrafish is an excellent model system to study thrombocyte function and development. Due to the difficulties in separating young and mature thrombocytes, comparative transcriptomics between these two cell types has not been performed. It is important to study these differences in order to understand the mechanism of thrombocyte maturation. Here, we performed single-cell RNA sequencing of the young and mature zebrafish thrombocytes and compared the two datasets for young and mature thrombocyte transcripts. We found a total of 9143 genes expressed cumulatively in both young and mature thrombocytes, and among these, 72% of zebrafish thrombocyte-expressed genes have human orthologs according to the Ensembl human genome annotation. We also found 397 uniquely expressed genes in young and 2153 uniquely expressed genes in mature thrombocytes. Of these 397 and 2153 genes, 272 and 1620 corresponded to human orthologous genes, respectively. Of all genes expressed in both young and mature thrombocytes, 4224 have been reported to be expressed in human megakaryocytes, and 1603 were found in platelets. Among these orthologs, 156 transcription factor transcripts in thrombocytes were found in megakaryocytes and 60 transcription factor transcripts were found in platelets including a few already known factors such as Nfe2 and Nfe212a (related to Nfe2) that are present in both megakaryocytes, and platelets. These results indicate that thrombocytes have more megakaryocyte features and since platelets are megakaryocyte fragments, platelets also appear to be thrombocyte equivalents. In conclusion, our study delineates the differential gene expression patterns of young and mature thrombocytes, highlighting the processes regulating thrombocyte maturation. Future knockdown studies of these young and mature thrombocyte-specific genes are feasible and will provide the basis for understanding megakaryocyte maturation.

## Introduction

Thrombocytes in fish and birds have been shown to be platelet equivalents and participate in hemostasis to prevent blood loss during an injury. We reported earlier that there are two types of thrombocytes in zebrafish, the young and mature thrombocytes [1–3]. The classification of these thrombocytes was by the fortuitous labeling of a group of thrombocytes at a specific concentration DiI-C18 (DiI) [3]. These thrombocytes were functionally more active and were first recruited to the injury site. Interestingly, even among the DiI labeled thrombocytes, there are the most intensely labeled and less intensely labeled which suggest a continuum of different stages of thrombocyte maturation. Also, we noted two types of thrombocytes in the Fli1-GFP transgenic zebrafish, the less intense GFP+ thrombocytes and more intense GFP+ thrombocytes. Moreover, the less intense GFP+ thrombocytes were DiI positive. Therefore, we concluded that less intense GFP+ thrombocytes are young thrombocytes. Surprisingly, these less intense GFP+ thrombocytes are also positive for RFP driven by the *mlc2* promoter [4]. Thus, the RFP+ thrombocytes are young, and the rest of the more intense GFP+ thrombocytes are shown to be mature thrombocytes, probably in different stages of maturation.

Megakaryocytes are precursors for human platelets, and they mature in the bone marrow [5]. These cells advance in maturation by endomitosis, yielding polyploidy [6, 7]. However, this feature appears to be unique to mammals. Interestingly from fish to birds, there are no megakaryocytes reported; however, thrombocytes seem to possess properties of megakaryocytes in at least having conserved transcription factors [8]. For example, the young thrombocytes, when they mature, gain more Fli1 with the concomitant loss of GATA-1 transcription factor [9]. We have also shown that there is a decrease in phosphatidylethanolamine (PE) and an increase in phosphatidylcholine (PC) in mature thrombocytes compared to young thrombocytes [4]. Interestingly, amphibian thrombocytes under *in vitro* conditions showed polyploidy-like features [10]. Several genes such as *CD41*, *GP1B*, *P-SELECTIN*, *ACVR1*, and *PAR1* are expressed in megakaryocytes and have been shown to be expressed in thrombocytes [11]. Thus, we have argued that thrombocytes have both megakaryocyte and platelet properties except for polyploidization. Despite these differences, the overall thrombocyte development could be used to study megakaryocyte development and maturation. Also, there are no comprehensive genetic studies to understand megakaryocyte development. At the best, we could study megakaryocyte development *in vitro* in cell cultures systems but will require animal models for confirming these studies. Thus, we believe that thrombocyte development and maturation in zebrafish could model megakaryocyte development maturation. However, such studies require a comprehensive analysis of factors that control thrombocyte development and maturation.

As an initial step to understanding thrombocyte maturation, we sought to compare the transcriptional profiles of the young and mature zebrafish thrombocytes. Our study shows that in the full collection of thrombocytes (young and mature), there were 9143 gene transcripts in contrast to 7708 gene transcripts in megakaryocytes and 2819 gene transcripts in platelets in the published databases. Our study also shows that there are 397 genes expressing uniquely in young thrombocytes and 2153 genes that expressed in mature thrombocytes only. We used the excel spreadsheet to eliminate the duplicates and found that among the 397 genes, 272 genes had human orthologs. Likewise, of the 2153 genes expressing only in mature thrombocytes, 1620 are orthologous to human genes. We have also compared megakaryocyte expression profiles with the thrombocytes and found that the thrombocytes and megakaryocytes have common transcripts for 4224 genes. Likewise, when we compared platelet expression profiles with thrombocytes, we found 1603 gene transcripts that are commonly expressed between thrombocytes and platelets. Interestingly, none of the unique genes expressed in young thrombocytes was found to be expressed in either megakaryocytes or platelets. However, among the unique gene transcripts found in mature thrombocytes, 602 were found in megakaryocytes, and 225 were found in platelets. We have also performed heatmap analysis, a listing of human orthologs, and provided information on select categories of genes. Thus, our analysis in this paper provides comprehensive information on the differential expression of genes in young and mature thrombocytes that should be useful to study the mechanism of thrombocyte maturation and might also be useful in understanding megakaryocyte maturation and development.

## Methods

### Zebrafish husbandry

Adult wild-type (WT) zebrafish and transgenic TG (fli1: EGFP) fish, Glo fish (DsRed), GloFli fish were maintained at 28°C in the circulating system of deionized water supplemented with Instant Ocean. The fish were maintained at 10 hours of dark and 14 hours of the light cycle and fed with brain shrimp and flakes [12].

### Zebrafish blood collection and cell sorting

The fish was placed on a clean paper towel on its side, and the skin was wiped gently with Kim Tech wipes. It was then clipped by using a pair of dissection scissors between the second and fourth black stripes in line with the ventral and the dorsal fins. 2 μL of blood was collected by using a 10 μL pipette, and 2 μL of blood was added into polystyrene 5ml conical tubes (BD Falcon, 12 x 75 mm), containing 300 μL of freshly prepared ice-cold 1x Phosphate buffered saline (PBS) supplemented with 0.04% of Bovine Serum Albumin (BSA) [13]. For cell sorting, GFP+ thrombocytes and RFP+ thrombocytes from GloFli fish were sorted using BD FACSCanto flow cytometer (UT Southwestern) separately into polystyrene 5ml conical tubes containing 1x PBS supplemented with 0.04% BSA. FACSCanto flow cytometer was set at 4°C with a nozzle size of 100. For optimum analysis, cell viability was set at 75% to 90% with at least 10,000 sorted cells. Samples run on FACSCanto flow cytometer were gated on PE and GFP using BD FACSdiva 8.0.2 software. For negative control, WT zebrafish were used, and for positive controls, Glo fish (DsRed) and TG (fli1: EGFP) fish were used. The sorted thrombocytes were kept on ice and were used immediately for RNA-seq by the Next Generation Sequencing Core at UT Southwestern.

### Humane care of zebrafish

Since anesthesia affects blood clotting, and fish blood generally clots faster than human blood, blood collection is very difficult. Thus, the blood collection must be performed without anesthesia. The animals used to collect blood will not survive, and therefore, they are euthanized immediately after blood collection by overdosing the fish with buffered MS222 (2%) anesthetic. All procedures were approved by the Institutional Animal Care and Use Committee (Protocol # 20021) of the University of North Texas, and animal experiments were performed with humane care in compliance with the institutional guidelines.

### RNA sequencing

RNA from sorted GFP+ thrombocytes and RFP+ thrombocytes were prepared according to 10x Genomics protocol. Sequencing was performed after successful library preparation and quality control. Briefly, the sorted cells were loaded immediately onto 10x Genomics Chromium Chip B. The barcoded cDNA libraries were generated using 10x Genomics Chromium Single Cell 3’ GEM library and Gel Bead Kits V3 according to the manufacturer’s instructions. The library sequencing on Illumina Hiseq 2500 V3 was performed on a depth of a minimum of 20,000 read pairs/cell for each library, and Cell Ranger software (3.0.0) version was used for barcode recovery [14–16].

### Analysis of duplicated genes in zebrafish and identifying human orthologs

We used the raw data and obtained the list of zebrafish ‘protein-coding’ genes for GFP+ thrombocytes and RFP+ thrombocytes using Ensembl and ZFIN databases. The list of the above genes was imported to an excel spreadsheet to analyze the duplicated genes. The GFP+ and RFP+ thrombocyte gene lists were colored green and red, respectively. To identify the common genes expressed in both GFP+ and RFP+ thrombocytes gene lists, we combined both lists and used the ‘conditional formatting’ function to highlight and then to find the duplicated values. We then removed the duplicated genes by using the ‘remove duplicates’ function to identify by their green and red color codes using the ‘sort and filter by color’ function, the unique GFP+ and RFP+ genes. After removing the duplicates from GFP+ and RFP+ genes, we used the list of human genes from the Ensembl database and performed a similar analysis to identify human orthologs that would appear as duplicates when zebrafish and human genes are combined.

### Gene classification

For gene function classification, Protein Analysis Through Evolutionary Relationship (PANTHER) (version 16.0) online software was used to classify the proteins encoded by genes of interest based on functional classifications, including family and subfamily, molecular function, cellular component, biological process, and pathway [17]. This method eliminates non-protein coding genes such as microRNA genes. The gene lists for each dataset, GFP+ thrombocytes, RFP+ thrombocytes, human megakaryocytes, and platelets, were entered with the gene IDs, and *Danio rerio* genome source ZFIN was chosen for analysis. The outputs of functional classification analysis were visualized using graphic charts and also presented as the summary of genes.

### Heat maps

Transcripts from single-cell RNA-seq data were quantified using Salmon [18]. The expression data that was generated through the 10x Genomics default pipeline was annotated using genes primarily taken from ZFIN. Gene groups were taken from BioMart-Ensembl for all available genes. The only group annotation in ZFIN was a basic group type (protein_coding, miRNA, etc.), but the actual gene function was not annotated. There are several genes in the expression files that do not have any entry in ZFIN. These genes begin with "LOC." If there was data in ZFIN or BioMart-Ensembl, they were included.

Genes were clustered based on their expression via k-means clustering ranging from *k* = 2 to *k* = 10. The expression of genes is based on the average mean counts of cells falling into the generated clusters. An additional graph-based clustering was also performed, which is independent of a *k*-value. In this case, 4 clusters were obtained. The cellRanger t-SNE graphs were used in choosing the best clustering. We then generated the spreadsheet with a sheet for each cluster type.

The counts covered a large range (0–120+), and row-wise scaling on the values was performed. This scaling provides a range of -2 to 2 such that everything will not be in the same color and two annotation bars covering the map. Due to the different number of genes in some of the sets, I row annotations were turned off. Row-wise re-ordering is based on an invisible hierarchical clustering of gene-wise distances. Column re-ordering was turned off to maintain the column order of the original excel data. Software used was the ’aheatmap’ function of the NMF library for R. Scaling was performed using the scale function of R with a transposed matrix of the mean counts.

### TPM analysis

Transcripts from single-cell RNA-seq data organized in 24 scRNASeq files were quantified using Salmon [18]. Among these 24 files in fastq format, reads from 8 files mapped to the zebrafish genome with a mapping percentage of almost 45–46%. Among these 8 files, 4 represented GFP+ thrombocytes, and the remaining represented RFP+ thrombocytes. Therefore, further downstream processing was done on these 8 files. Since the 4 fastq files were derived from the same GFP+ thrombocytes, we merged them to generate a single GFP+ thrombocyte file. Likewise, we generated a single file for RFP+ thrombocytes.

Transcripts per million (TPM) distribution obtained in this analysis was divided into arbitrarily five intervals. For each of the GFP+ and RFP+ thrombocyte datasets, we classified genes as expressed when their TPM values were higher than 1. Gene-level abundance estimates were obtained using the R package (tximport) [19].

### Quantile mapping

We have divided the expressed genes in each dataset into 10 quantiles based on their expression levels, i.e., the first quantile consists of the top 10% (approximately) of the expressed genes in the dataset, arranged in decreasing order of their expression values, etc. We performed quantile mapping by comparing the genes in each quantile across a pair of datasets. The naming design was GFP_x_RFP_x_Genes; here, x ranges from 1 to 10. For example, GFP_9_RFP_5_Genes denotes genes in the 9th quantile of GFP that overlapped with genes in the 5th quantile of RFP.

### System biology analysis

To identify functional similarity in the two datasets (GFP+ thrombocytes and RFP+ thrombocytes), we performed a comparative analysis at the systems level. For this purpose, we identified “highly expressed” genes in each dataset as the genes that have a z-score (based on log TPM values of “expressed” genes) greater than 1.5 in each dataset. The highly expressed gene lists for GFP+ and RFP+ thrombocytes were analyzed separately using the database for annotation, visualization, and integrated discovery (DAVID V6.8) to identify enriched Kyoto encyclopedia of genes and genomes (KEGG) pathways, and gene ontology (GO) categories, namely, the biological process (BP), cellular component (CC), and molecular function (MF). The criterion of FDR (False Discovery Rate) less than or equal to 0.05 (in DAVID, FDR of 5 is equivalent to 0.05) was used to identify enriched GO terms and KEGG pathways. In DAVID, the FDR values are provided as FDR percentage, so, FDR = 1 means an FDR of 1/100 = 0.01 [19].

### Megakaryocyte and platelet RNA-seq analysis

We used megakaryocyte single-cell RNA-seq dataset GSM4278189, which was downloaded from NCBI GEO. There were 4 fastq files derived from the same sample, which were merged to generate a single fastq file. For platelets, we used platelet RNA-seq dataset SRP262885, which was downloaded from NCBI SRA. There were 5 fastq files derived from the 5 different samples. These fastq files (1 for megakaryocyte and 5 for platelets) were further processed and transcript abundance quantified using Salmon, and the gene-level abundance estimates were obtained using the R package (tximport). We identified genes as “expressed” in a dataset if their TPM values were higher than 1. We identified “highly expressed” genes in each dataset as the genes that have a Z-score (based on log TPM values of “expressed” genes) greater than 1.5 in the dataset.

## Results

### Gene expression in young and mature thrombocytes

To understand the genes involved in the maturation of thrombocytes, we performed RNA-seq analysis of young and mature thrombocytes and studied conserved and differential gene expression patterns between these thrombocytes. We used the GloFli adult zebrafish, where young thrombocytes were labeled with RFP, and mature thrombocytes were labeled with more intense GFP. This adult zebrafish line, GloFli, was previously characterized in our laboratory [4]. We collected 2 μL of blood from a single fish and subjected this to flow cytometry for separating RFP+ and GFP+ cells. Attempts to pool blood from multiple fish yielded mostly dead cells because of the time taken from sorting to the loading on the 10x genomics machine. We used 1,541 GFP+ mature thrombocytes and 2,176 RFP+ young thrombocytes for single-cell RNA sequencing (scRNA-seq). The quality of cDNA from GFP+ and RFP+ thrombocytes used for sequencing is shown in S1 Raw images. The raw data are given in Data Review URL (https://dataview.ncbi.nlm.nih.gov/object/PRJNA753153?reviewer=l15a7d4am3m124rafh16mbsplv). We used these raw data files and obtained human orthologs and the corresponding human genes and respective genetic disorders associated with them using Ensembl and ZFIN databases. This reorganized data is shown in S1 and S2 Files. From this data, we found the total number of genes detected (genes with transcripts) for GFP+ cells is 8,746 and for RFP+ cells is 6,990 genes. By combining the datasets from GFP+ and RFP+ thrombocytes and after eliminating the duplicated genes, 6,593 genes were found to be commonly expressed in both GFP+ and RFP+ thrombocytes, thus, yielding a total of 9143 genes expressed cumulatively in young and mature thrombocytes. Among these genes expressed in both GFP+ and RFP+ thrombocytes, we found CD41 gene transcripts [20]. Also, we found that mature thrombocytes uniquely expressed 2,153 genes, young thrombocytes expressed 397 genes exclusively by our spreadsheet method.

### Protein coding genes

**Fig 1:**
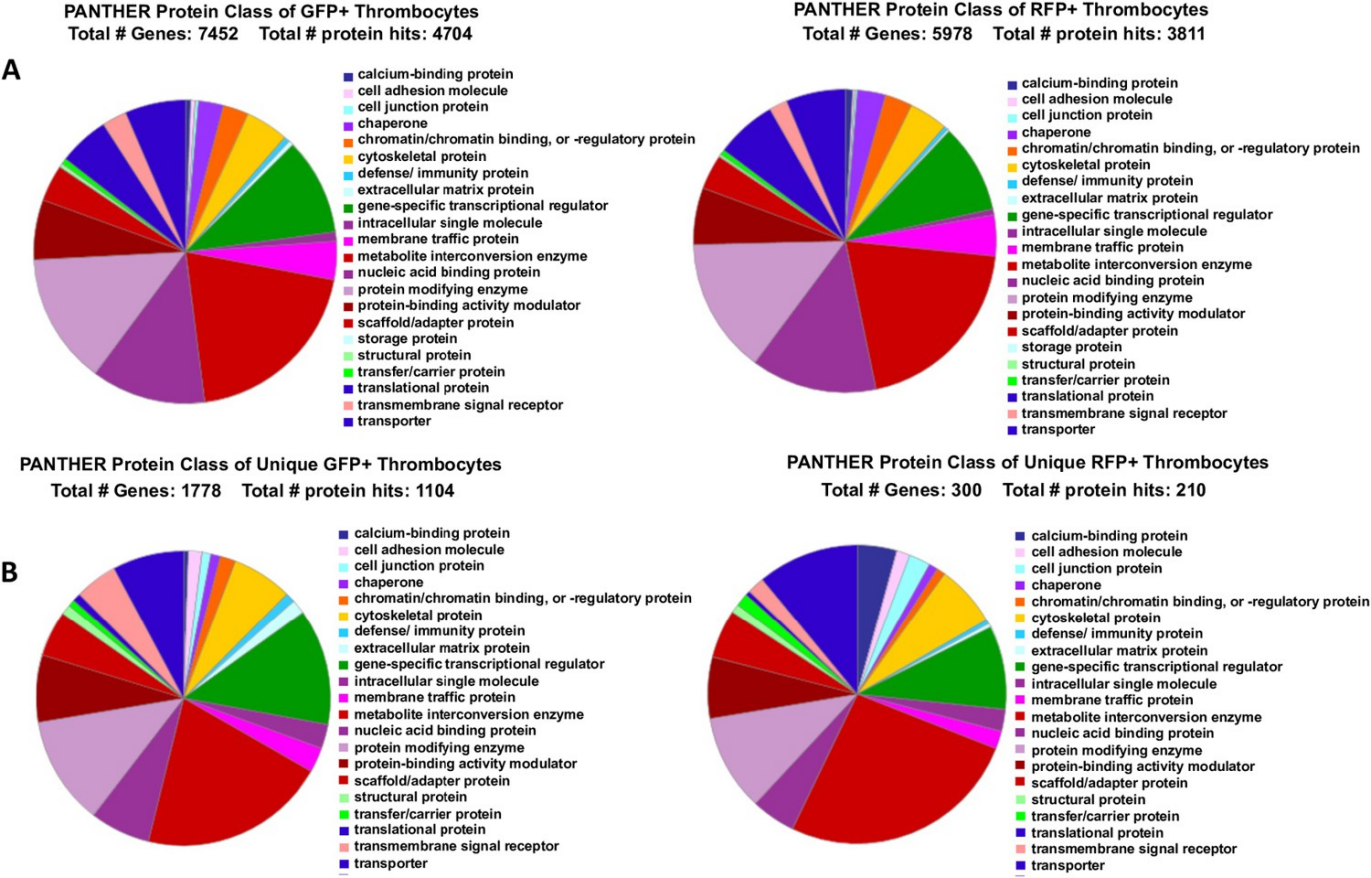
PANTHER analysis and classification of GFP+ and RFP+ thrombocyte transcripts. A. When 8,746 genes expressed in GFP+ and 6990 genes expressed in RFP+ thrombocytes thrombocytes were uploaded into the PANTHER program, and *Danio rerio* species was selected, GFP+ 7452 genes and RFP+ 5978 genes, respectively were detected by this program. The program yielded a total number of protein hits of 4704 and 3811 for these GFP+ and RFP+genes, respectively. B. When 2153 unique genes expressed in GFP+ thrombocytes and 397 unique genes expressed in RFP+ thrombocytes were uploaded into the PANTHER program, and *Danio rerio* species was selected, GFP+ 1778 genes and RFP+ 300 genes, respectively, were detected by this program. The program yielded a total number of protein hits of 1104 and 210 for these unique GFP+ and RFP+unique genes, respectively. In both A and B, the percentage of each of the protein classes for GFP+ and RFP+ gene lists are shown by different colors in the pie chart, and their classification is provided by its side with similarly coded colors.

To eliminate the non-protein coding genes such as microRNA genes, the expressed genes from GFP+ (8,746) and RFP+ (6,990) thrombocytes were functionally characterized using the PANTHER online software that performs protein-coding gene functional classification. The protein gene classes for GFP+ and RFP+ thrombocytes are given in Fig 1 as a pie-chart. The number of genes detected (genes with transcripts) for GFP+ cells by PANTHER analysis was 7,452 and for RFP+ cells was 5978. However, the protein coding genes in the GFP+ and RFP+ thrombocytes were 4704 and 3811, respectively. When we analyzed the uniquely expressed genes in the GFP+ thrombocytes by PANTHER, we found 1,778 and 300 genes had transcripts in GFP+ and RFP+ thrombocytes, respectively. However, the unique protein genes in GFP+ and RFP+ thrombocytes were 1,104 and 210, respectively.

### Heat maps

**Fig 2:**
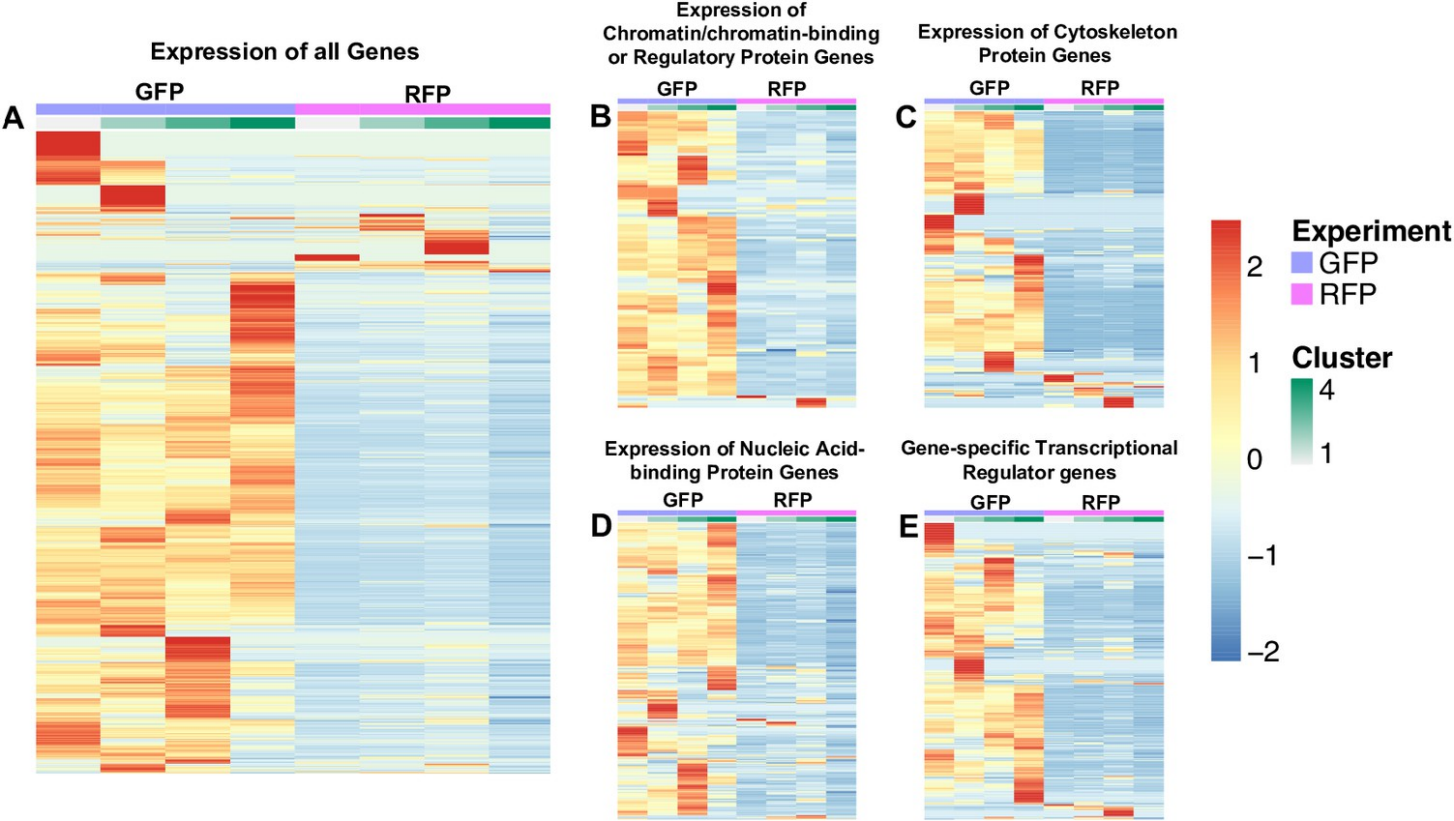
Heat map of differentially expressed genes between GFP+ and RFP+ thrombocytes. Genes were arbitrarily clustered via k-means clustering ranging from k = 2 to k = 10. The expression of genes is based on the average mean counts of cells falling into the generated clusters. The cellRanger t-SNE graphs were used in choosing the best clustering and 4 clusters were found. A spreadsheet with a sheet for each cluster type was generated. The counts covered a large range (0–120+), and row-wise scaling on the values was performed, which provided a range of -2 to 2. A. Expression of all genes, 8746 genes in GFP+ thrombocytes and 6990 genes in RFP+ thrombocytes. B. Expression of chromatin/chromatin-binding or regulatory protein genes, 120 genes in GFP+ thrombocytes and 106 genes in RFP+ thrombocytes. C. Expression of cytoskeleton protein genes, 217 genes in GFP+ thrombocytes and 158 genes in RFP+ thrombocytes. D. Expression of nucleic acid-binding protein genes, 535 genes in GFP+ thrombocytes, and 478 genes in RFP+ thrombocytes. E. Expression of gene-specific transcriptional regulator genes, 449 genes in GFP+ thrombocytes, and 329 genes in RFP+ thrombocytes. Purple indicates GFP+ thrombocytes, and pink indicates RFP+ thrombocytes followed by clusters from 1 to 4 shown in light green to dark green. For each gene, red is upregulated, and blue is downregulated in the corresponding sample.

Using the transcripts from RNA-seq data, the heatmaps of the gene expression for GFP+ and RFP+ thrombocytes were generated, and they are shown in Fig 2. The heat maps showed that the highly expressed genes in the RFP+ population are limited. The list of genes used in this heat map study is given in S3 File. We also found that highly expressed genes among certain categories, such as chromatin/chromatin-binding or regulatory protein genes, cytoskeleton protein genes, nucleic acid-binding protein genes, and gene-specific transcriptional regulator genes are less in the RFP+ population compared to the GFP+ thrombocyte population.

### TPM analysis

**Fig 3:**
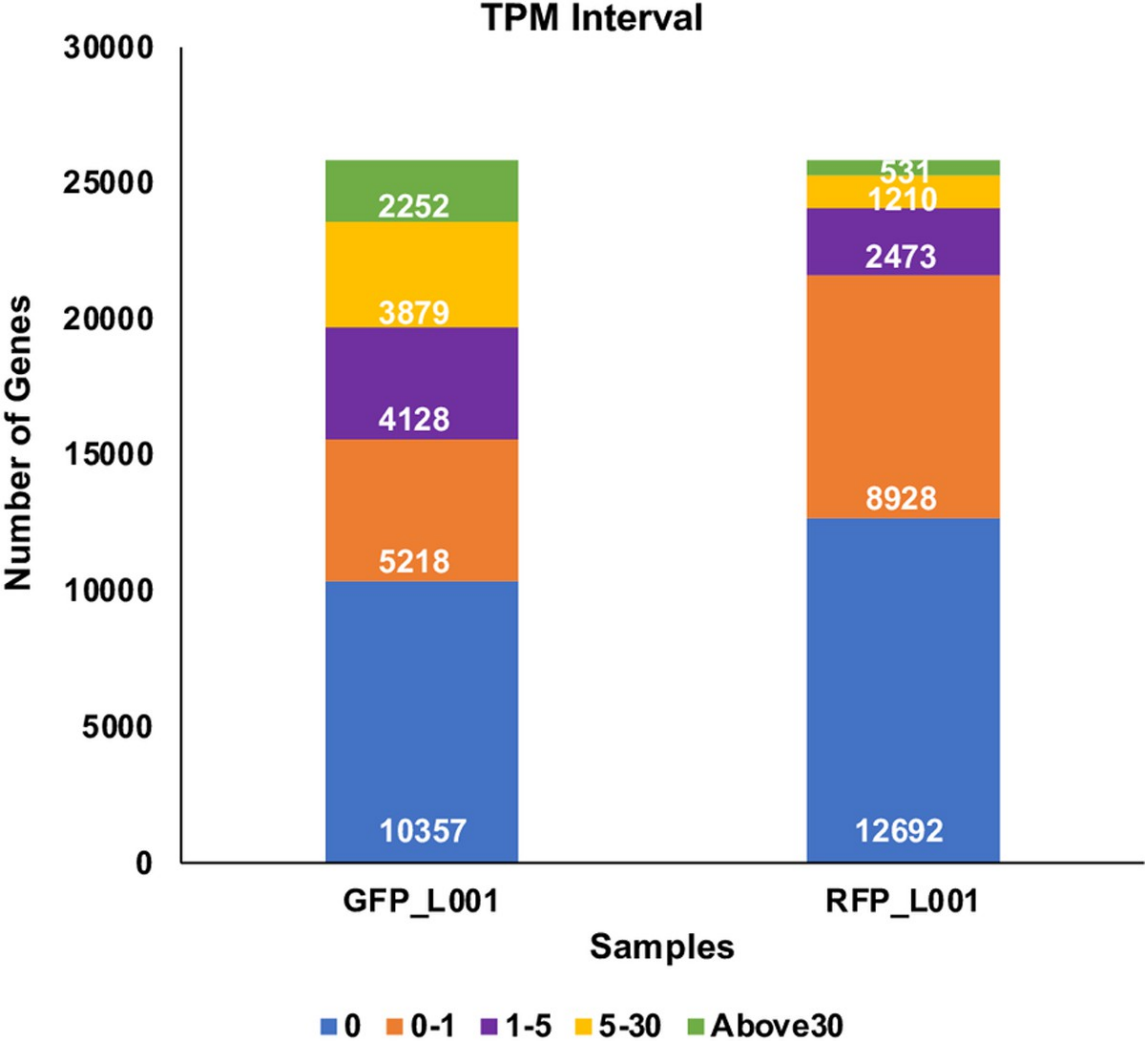
Transcripts per million (TPM) distribution for each of the two samples GFP_L001 and RFP_L001 representing GFP+ and RFP+ thrombocytes. Transcripts from 24 scRNASeq files, 12 for GFP+ and 12 for RFP+ thrombocytes were quantified. Among these files, 4 GFP+, and 4 RFP+ files (in fastq format) that showed a mapping percentage of almost 45–46% to the zebrafish genome were chosen. The 4 GFP fastq files were merged to one fastq file, and similarly 4 RFP fastq files were also merged to one fastq file and used in TPM analysis. Bar graphs show TPM distribution obtained in this analysis and are divided into five intervals for each sample representing the number of genes with TPM values from 0 to >30, and these TPM values are coded by different colors with a key shown below.

We obtained the gene-level abundance estimates using the R package using two merged scRNASeq files, one for GFP+ and the other for RFP+ thrombocytes. The TPM values for each of the two merged scRNASeq files are provided in S4 File. We then analyzed the TPM distribution and the results of this distribution, for each of the two samples (GFP+ and RFP+ thrombocytes) used in our analysis are given in Fig 3. Genes expressing with a TPM value greater than 1 in GFP+ and RFP+ thrombocytes are enlisted in S5 and S6 Files (“expressed genes”). To identify functional similarities in the two datasets (GFP+ and RFP+), we performed a comparative analysis of gene expression at the systems level. For this purpose, we identified “highly expressed” genes in each dataset as the genes that have a Z-score (based on log TPM values of “expressed” genes) greater than 1.5. The log TPM values for GFP+ and RFP+ datasets are provided in S7 and S8 Files. The z-scores of all genes expressed in GFP and RFP thrombocytes are given in S9 and S10 Files. This resulted in 867 “highly expressed” genes for GFP+ and 302 for RFP+ cells. The list of highly expressed genes in these two thrombocyte populations is given in S11 and S12 Files. The results also revealed 265 highly expressed genes commonly present in both these thrombocyte populations. Thus, most of the genes analyzed showed very low or no expression. On average, 40.09% and 49.12% of genes had zero TPM for GFP+ and RFP+ cells, respectively. We also found that 20.19% and 34.55% of genes had less than one TPM in GFP+ thrombocytes and RFP+ thrombocytes, respectively. We found 15.94% and 9.57% of genes had TPM between the interval of 1–5 in GFP+ and RFP+ thrombocytes, respectively. We also found 15.01% and 4.68% of genes had TPM between the interval of 5–30 in GFP+ and RFP+ thrombocytes, respectively. The result also revealed 8.71% of GFP+ genes had a TPM of 30 and higher, contrasting with 2.05% of such RFP+ genes. Interestingly, there was a 3–4 folds increase in the number of genes with TPM values greater than 5 between the RFP+ and GFP+ thrombocytes.

### Quantile mapping

**Fig 4:**
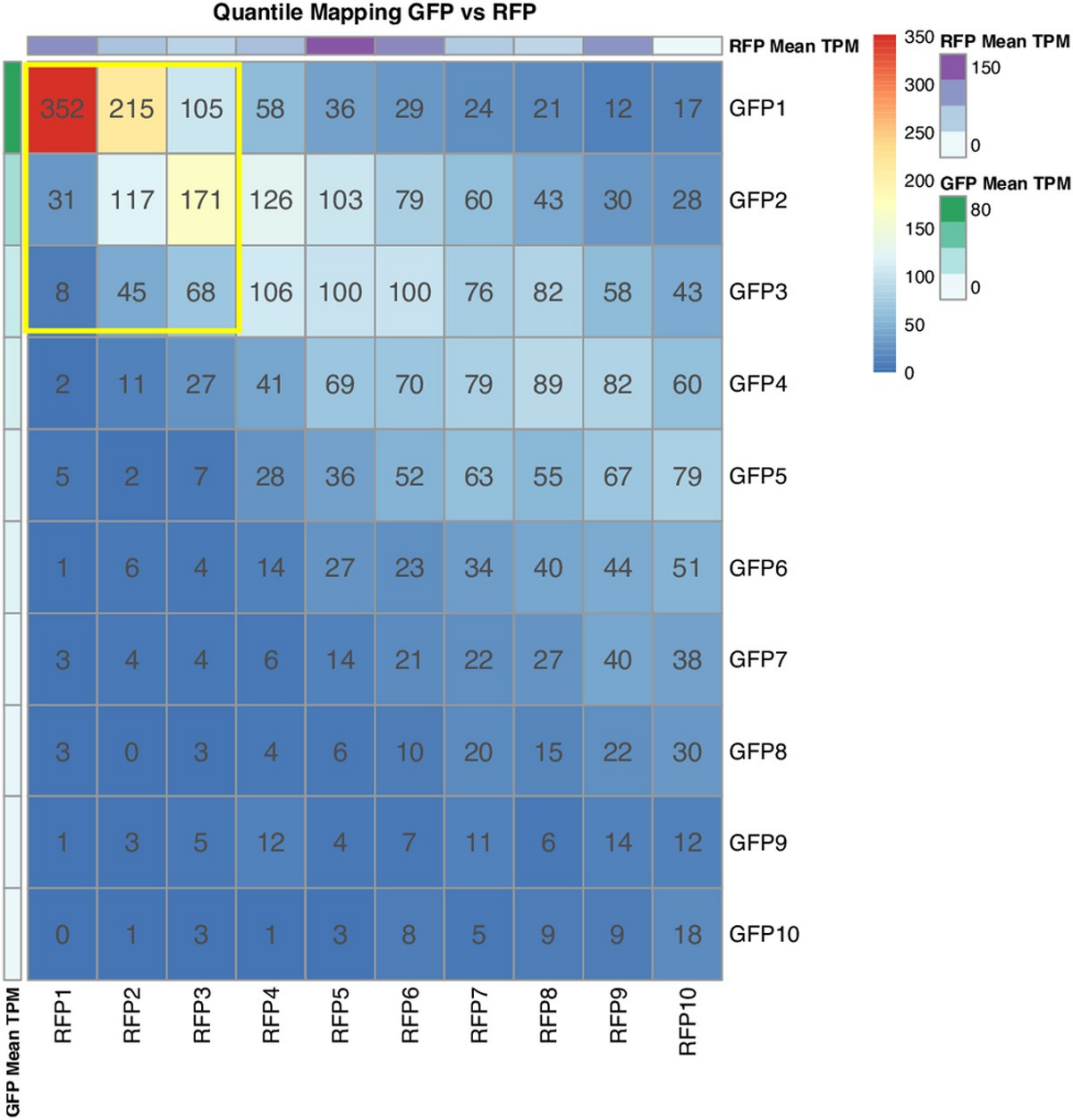
Quantile mapping of GFP+ thrombocyte and RFP+ thrombocyte datasets. We have divided the expressed genes in each dataset into 10 quantiles. We have compared the genes in each quantile across the pair of datasets. In each quantile, the quantile map shows the number of common genes between the two datasets. The bars on the right of the quantile map indicate the color codes and the number of genes. The data value bars on the rightmost indicate mean TPM values; green color, the highest TPM values for GFP+ thrombocytes, and purple color, the highest TPM values for RFP+ thrombocytes.

The quantile-mapping analysis helped us identify the percentage of overlapping genes displaying similar expression patterns in GFP+ and RFP+ thrombocytes. The most highly expressed genes in the first quantile (top 10% in expression) for GFP+ and RFP+ thrombocytes have 352 genes common between them. The mapping results for each quantile pairs for GFP+ and RFP+ thrombocytes are provided in Fig 4. The details of quantile mapping are shown in S13 File. The overlapping genes between GFP+ and RFP+ thrombocytes in the other 9 quantiles are 1112, approximately 28% of 3935, the total number of genes in all the quantiles (Fig 4).

### Data set characterization

**Fig 5:**
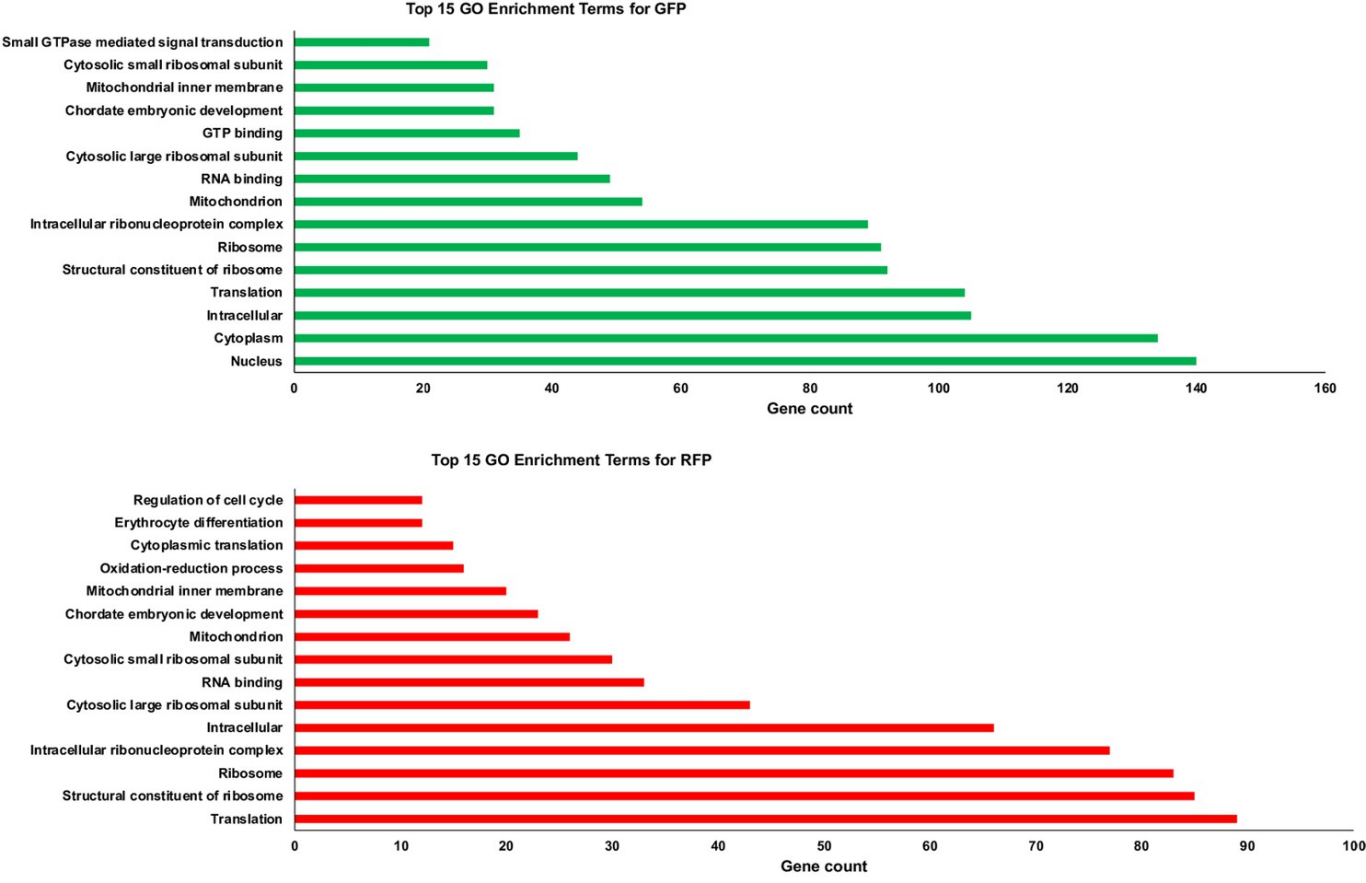
Gene ontology analysis of GFP+ and RFP+ thrombocyte transcripts. The highly expressed gene lists for GFP+ and RFP+ thrombocytes were analyzed separately using DAVID to identify gene ontology (GO) categories. The top 15 GO enrichment analysis of A. GFP+ thrombocytes and B. RFP+ thrombocytes by DAVID functional analysis. Bars represent the number (x-axis) of highly expressed genes in pathways (y-axis) for each dataset.

Further, we analyzed highly expressed genes list in the data sets for both GFP+ mature and RFP+ young thrombocytes. The DAVID program was used to identify KEGG pathways and functional terems representing GO categories, namely, the BP, CC, and MF, for each dataset. The top 15 GO enrichment terms for GFP+ and RFP+ thrombocyte datasets are shown in Fig 5. FDR values are provided in S14 and S15 Files. Interestingly, the GO analysis revealed 241 and 251 genes involved in ribosomal machinery of RFP+ and GFP+ thrombocytes, respectively, representing a modest 4.14% increase in the number of ribosomal machinery genes transcriptionally activated in the course of development from RFP+ to GFP+ thrombocytes. However, for the intracellular component, the RFP+ thrombocytes had 66 expressed genes, whereas the GFP+ cells had expressed 105 genes, representing 59% increase in the number of transcriptionally activated genes from RFP+ to GFP+ thrombocytes. The GO enrichment values for GFP+ and RFP+ thrombocytes are shown in S14 and S15 Files. The list of the top 15 GO enriched gene numbers is given in S16 File.

**Fig 6:**
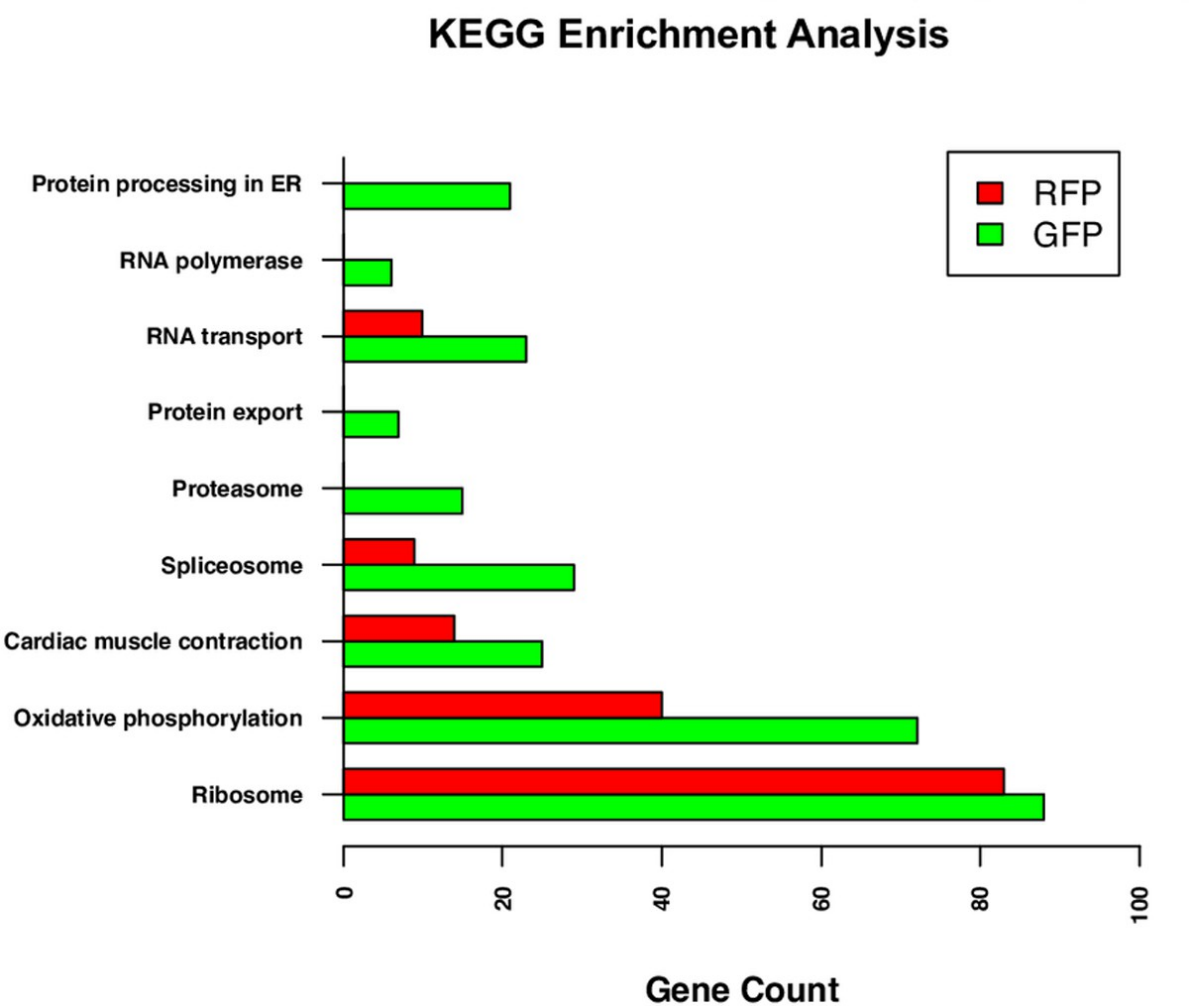
Significantly enriched Kyoto encyclopedia of genes and genomes. The highly expressed gene lists for GFP+ and RFP+ thrombocytes were analyzed separately using the DAVID to identify enriched KEGG pathways. Bars represent the number of highly expressed genes in pathways for GFP+ and RFP+ thrombocyte datasets.

The KEGG analysis yielded 9 datasets of the most highly expressed genes, each in GFP+ and RFP+ thrombocytes. The KEGG pathway enrichment analysis results are shown in Fig 6. The values for pathway enrichment for each dataset are provided in S17 and S18 Files. Except for the ribosome-related genes which are similar in numbers between GFP+ and RFP+ thrombocytes, the rest of the 8 datasets yielded greater number of genes expressed in GFP+ thrombocytes compared to RFP+ thrombocytes. Interestingly, we found erythrocyte differentiation genes expressed only in RFP+ thrombocytes.

### Human orthologs expressed in thrombocytes

Zebrafish genes that are expressed in thrombocytes were compared with human genes in the Ensembl database, and the orthologous human genes expressed in both young and mature thrombocytes were identified. They were found to be 7081 genes out of the 9143 genes (72%) expressed cumulatively in two types of thrombocytes according to Ensembl and ZFIN databases. However, since many of the zebrafish genes are duplicated, for each of these genes same human homolog will be picked up and thus will yield more duplicates in the human gene set. We, therefore, corrected for 460 human genes that were shown as duplicates or triplicates, yielding 6621 true human orthologs. Thus, approximately 72% of zebrafish genes expressed in thrombocytes are orthologous to human genes.

**Fig 7:**
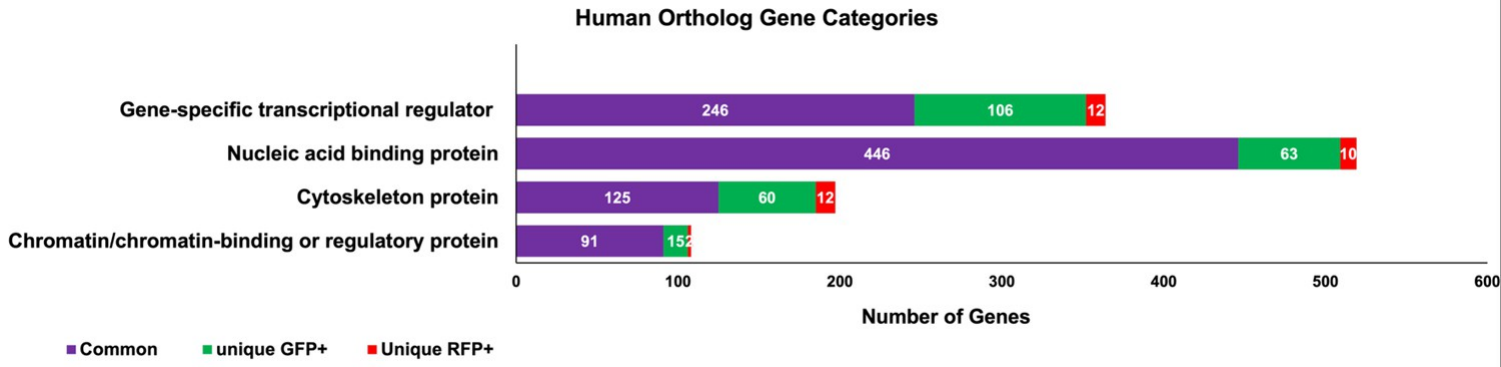
Different categories of human ortholog gene transcripts found in GFP+ and RFP+ thrombocytes. The raw data was used to obtain the list of zebrafish genes expressed in GFP+ thrombocytes and RFP+ thrombocytes using Ensembl and ZFIN databases. The list of human orthologs corresponding to zebrafish genes was also obtained using Ensembl and ZFIN databases. The list of these human orthologs were uploaded into the PANTHER program to obtain protein categories as well as to find human orthologs corresponding to unique and commonly expressed genes in GFP+ and RFP+ thrombocytes. The bar graph represents the corresponding human orthologs of the unique RFP+ genes, unique GFP+ genes, and both RFP+ and GFP+ common genes shown in red, green, and purple colors, respectively. The number of human ortholog genes is shown in each bar, including their functional protein categories. The protein categories are gene-specific transcriptional regulators, nucleic acid-binding proteins, cytoskeleton proteins, and chromatin/chromatin-binding or regulatory proteins.

Since we are interested in genes that are regulatory for thrombocyte development, we chose 4 categories of genes analyzed in heat maps such as chromatin/chromatin-binding regulatory protein, cytoskeleton, nucleic acid binding, and gene-specific transcriptional regulator genes. We first obtained the number of genes, each in GFP+ and RFP+ thrombocytes, that have human orthologs in the above 4 categories. We then determined the orthologs of the genes that uniquely expressed in GFP+ and RFP+ thrombocytes, as well as those that expressed across both these thrombocyte populations. The results are shown in Fig 7. The results revealed approximately 30% of the orthologs belong to these 4 categories in both GFP+ and RFP+ thrombocytes.

### Comparison of gene expression between thrombocytes and megakaryocytes/platelets

To test whether thrombocytes have similar transcripts found in megakaryocytes and platelets, we compared the transcripts present in thrombocytes obtained by our single-cell RNA-seq analysis with the transcripts derived from publicly available single-cell RNA-seq data from megakaryocytes from early human embryonic cells. We obtained a list of genes that are common in both datasets and designated the human counterparts as human orthologs. The list of orthologs of thrombocyte transcripts found in megakaryocytes are given in S19 and S20 Files and for platelets in S21 and S22 Files. We found 4224 out of 9143, i.e. 46% gene transcripts from thrombocytes to be present in the megakaryocytes. Likewise, when we compared with the platelet transcripts, we found 1603 out of 9143, i.e. 17% thrombocyte gene transcripts were in platelets. When we compared both megakaryocyte and platelet transcripts with the transcripts present in thrombocytes, we found 1339 gene transcripts common to all three. However, out of the unique 397 RFP+ gene transcripts, none of them was present in megakaryocytes or platelets, while of the unique 2153 GFP+ gene transcripts, 602 were present in megakaryocytes, whereas 225 were present in platelets. Interestingly, we found that among the 397 unique RFP+ thrombocyte gene transcripts, 272 had human orthologs. Likewise, out of 2153 unique GFP+ thrombocyte gene transcripts, 1620 had human orthologs.

**Fig 8:**
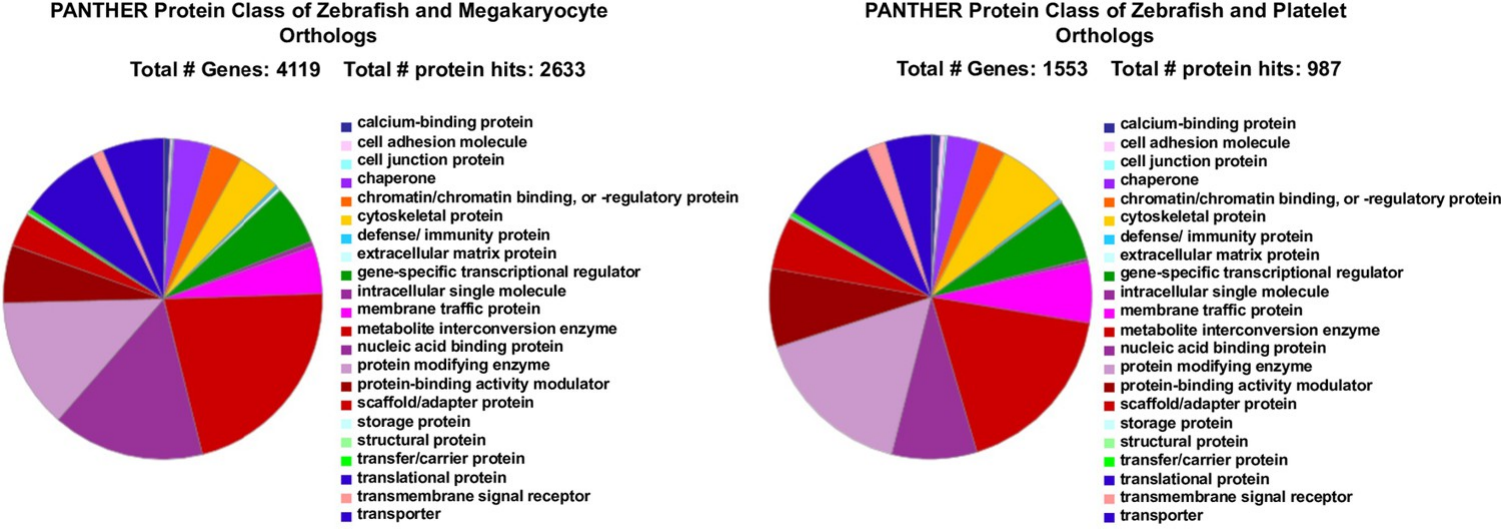
PANTHER analysis and classification of human orthologs of total thrombocyte gene transcripts found in human megakaryocytes and platelets. A. The 9143 total thrombocyte gene transcripts and 7708 human megakaryocyte gene transcripts were combined in a spreadsheet and color-coded. Then duplicate genes were selected first and the duplicates were eliminated to give a list of 4224 human orthologs which were uploaded into the PANTHER program, and *Danio rerio* species was selected. The program detected 4119 genes and yielded a total number of 2633 protein hits for these genes. B. The 9143 total thrombocyte gene transcripts and 2819 human platelet gene transcripts were combined in a spreadsheet and color-coded. Then duplicate gene transcripts were selected first, and the duplicates were eliminated to give a list of 1603 gene transcripts for human orthologs, which were uploaded into the PANTHER program, and *Danio rerio* species was selected. The program detected 1553 genes and yielded a total number of 987 protein hits for these genes. In both A and B, the percentage of each of the protein classes for RFP+ and GFP+ gene lists are shown by different colors in the pie chart, and their classification is provided by its side with similarly coded colors.

We have also analyzed the protein-coding transcripts using the PANTHER program and found several classes of proteins, grouped by functions, that are conserved between thrombocytes and megakaryocytes/platelets (Fig 8). From these data, we have chosen three families of proteins (transcription factors, cell adhesion molecules, and transmembrane receptors) involved in megakaryocyte/platelet development and function. We found a total of 156 transcription factor transcripts that are present in megakaryocytes. Among these, there were transcripts for 14 basic helix-loop-helix transcription factors, 16 basic leucine zipper transcription factors, 7 zinc finger transcription factors, 24 C2H2 zinc finger transcription factors, 7 C4 zinc finger nuclear receptors, 9 homeodomain transcription factors, 4 Rel homology transcription factors, and 9 wing helix/forkhead transcription factors. Among the basic leucine zipper transcription factors, Nfe2, an important factor for megakaryocyte development, is conserved.

We also found transcripts for a total of 6 cell adhesion molecules such as 4 integrins (Itfg2, Itgb3b, Itgb1a, Itga2b), Spondin domain-containing protein (Spon2a), and Thy1 cell surface antigen (Thy1). In addition to these two groups of transcripts, we also found transcripts for 9 GPCRs, including thrombin receptor (Par1) and transcripts for 19 transmembrane receptors, including erythropoietin and thrombopoietin receptors.

In thrombocytes, we also found transcripts for 60 transcriptional regulators that are also present in platelets. Among these, there were transcripts for 5 basic helix-loop-helix transcription factors, 4 basic leucine zipper transcription factors, 13 C2H2 zinc finger transcription factors, 1 C4 zinc finger nuclear receptors, 5 homeodomain transcription factors, 1 Rel homology transcription factor, and 4 wing helix/forkhead transcription factors. There were no zinc finger transcription factor transcripts among the platelet-specific transcripts. Interestingly, among the transcripts for 5 basic helix-loop-helix transcription factors found in thrombocytes, 4 of them were also present in megakaryocytes, whereas the *arnt* transcript was only found in platelets. Among the transcripts of basic leucine zipper transcription factors NFE2, an important factor for megakaryocyte development, is conserved. Likewise, another related transcript *nfe2l2a* was also shared among thrombocytes, megakaryocytes, and platelets. We found transcripts for 5 cell adhesion molecules such as 4 integrins (Itgb3b, Itgb5, Itgb1a, Itga2b), and Dag1. We did not find transcripts for Spon2a and Thy1. Likewise, we did not find transcripts for Itgb3b and Itgb5 in megakaryocytes. Moreover, we found transcripts for 7 GPCRs, including the one for Par1 that is also a megakaryocyte-specific transcript. However, two of these receptor transcripts *adora2ab*, *ipar5a* were found in platelets but not in megakaryocytes. Among the transcripts for 11 transmembrane receptors, both erythropoietin and thrombopoietin receptors’ transcripts were found similar to those in megakaryocytes.

## Discussion

Previous results from our laboratory, using microarray analysis, revealed thrombocyte-specific genes; however, there was no analysis of GFP+ and RFP+ thrombocyte-specific genes [21]. The work presented here is the first comparative analysis of GFP+ and RFP+ thrombocyte transcripts obtained by RNA-seq performed by 10x genomics. In this protocol, mRNA molecules were directly captured by Gel Bead oligo and, therefore, contaminated genomic DNA were not captured downstream. Also, we set up 75–90% cell viability in 10x genomics sequencing. We do not know whether this will skew the downstream data, and it is difficult to predict. However, bioinformatically there are steps that are usually taken to make sure that dead/dying cells are filtered out so that they do not influence the results of differential gene expression analysis. These QC or pre-processing steps usually involve filtering out cells with genes of too high expression or genes with too low expression due to possible background. They also filter out cells with a high percent of mitochondrial gene expression due to possible dead/dying cells. This procedure of filtering before further processing the data ensures that the downstream results reflect the biology of the system under investigation rather than that of cell death caused by the sample handling or preparation. Moreover, since in both of these populations, gene encoding CD41, a highly specific thrombocyte marker, was found to be expressed as revealed in the analysis of RNA-seq data obtained from the sorted populations of thrombocytes, it lends credibility to the thrombocyte populations and confirms that they are indeed thrombocyte populations with minimal contaminations from other blood cells.

The comparative analysis of the GFP+ and RFP+ thrombocytes showed, more highly expressed genes in GFP+ thrombocytes compared to RFP+ thrombocytes, as confirmed by the heat map studies, probably because during maturation more number of genes are turned on while less number of genes are silenced. In our previous work on young and mature thrombocytes, we have shown that there was an increase in the expression of a Fli1 in mature thrombocytes [1, 4]. These results are consistent with the heatmap results suggesting that maturation of thrombocytes involves an increase in thrombocyte gene expression [4]. The number of genes analyzed using excel spreadsheet analysis tools is larger than the ones obtained by PANTHER analysis and does not match because there are several genes that are not recognized by the PANTHER program. This may be due to the reason that the database used may not contain all the genes. In fact, the TPM analysis also showed there are four times more genes that are highly expressed in GFP+ thrombocytes compared to RFP+ thrombocytes.

Understanding thrombocyte maturation involves a closer look at regulatory genes involved in chromatin modification, transcriptional regulation, cytoskeletal reorganization, and nucleic acid-binding. Interestingly, the number of unique genes in RFP+ thrombocytes is less compared to GFP+ thrombocytes. These results are consistent with the total number of highly expressed genes and again suggest that the loss in expression of a fewer number of genes among these groups and the gain in expression of a greater number of genes are important for thrombocyte maturation. Previously it has been shown in our laboratory that there was a switch of a synthetic pathway from PE to PC. Thus, the current finding of the unique genes in GFP+ and RFP+ thrombocytes adds to the knowledge in understanding the regulation of overall thrombocyte maturation.

Our analysis by quantile mapping on the level of gene expression between GFP+ and RFP+ thrombocytes also revealed a smaller percentage of genes that are highly expressed between these cells, and a greater number of genes that are commonly expressed at a lower level. These results suggest that the highly expressed genes may be performing housekeeping functions and the genes that are expressed in lower levels may perform specialized thrombocyte function and development. Interestingly, GO studies also showed housekeeping genes such as those coding intracellular group of proteins and the proteins involved in ribosomal machinery are highly expressed in both GFP+ and RFP+ thrombocytes, and genes encoding specialized proteins such as those involved in erythrocyte differentiation are expressed at lower levels, consistent with quantile mapping. KEGG analysis revealed certain specialized functions such as proteosomes, spliceosomes, protein export, and protein processing in ER to be uniquely represented in GFP+ thrombocytes, suggesting that these specialized functions are important and necessary for thrombocyte maturation.

Our finding of 72% human orthologs among the zebrafish thrombocyte transcripts is strikingly similar to the percentage observed by the authors of the zebrafish genome project [22]. In addition, a comparison of expressed genes between thrombocytes and megakaryocytes/platelets revealed an important fact that thrombocytes share many transcripts with megakaryocytes and platelets. In fact, the finding of a greater number of orthologs of thrombocyte genes expressing in megakaryocytes compared to platelets suggests that thrombocytes have more megakaryocyte features so far as the transcripts are concerned relative to platelets and are probably megakaryocyte equivalents, and since platelets are fragments of megakaryocytes, thrombocytes also possess the properties of platelets. The higher number of transcript similarities to megakaryocytes relative to platelets is probably because platelet mRNAs, although derived from megakaryocytes may not be stable due to the lack of complete cell architecture in platelets, again indicating thrombocytes have cell machinery similar to megakaryocytes. These results suggest that thrombocytes are forerunners for megakaryocytes where polyploidization occurred at the time of mammalian radiation. Since the thrombocytes carry necessary components for aggregation and can be released into circulation, they perform platelet functions. This finding thus supports our previous hypothesis that thrombocytes have both megakaryocyte and platelet properties [23]. This fact is also strengthened by our finding that transcripts for transcription factors that are important for the differentiation and maturation of megakaryocytes are present in thrombocytes, and the platelets may carry a small number of transcripts derived from megakaryocytes. Likewise, the functionally important cell adhesion molecule transcripts are present in megakaryocytes, platelets, and thrombocytes.

It is well known that megakaryocytes are derived from a bifunctional erythrocyte-megakaryocyte precursor. Thus, young thrombocytes may carry certain properties of erythrocytes, and our results are consistent with this megakaryocyte differentiation. This assumption is supported by the observation that expression of erythrocyte receptor genes occurs in young thrombocytes. Also, young thrombocytes express Gata1, an erythroid transcription factor [24, 25]. Surprisingly, among the unique genes expressed in young thrombocytes, none of them was found in megakaryocyte transcripts, probably because the megakaryocyte RNA-seq data derived from human embryonic cells may not have representation of younger megakaryocytes. Thus, these unique genes are probably novel and important for thrombocyte maturation.

In summary, the above comparative analysis revealed several genes that may be involved in the maturation of thrombocytes. These genes could be used in analyzing their function in thrombocyte maturation by gene knockdown studies. For example, the knockdown of unique RFP+ genes may advance thrombocyte maturation. In contrast, the knockdown of unique GFP+ genes may stop the maturation. The comparison of thrombocyte transcripts with megakaryocyte/platelet transcripts revealed that thrombocytes are both megakaryocyte and platelet equivalents.

## Conclusions

In conclusion, our single-cell RNA-seq analysis and comparison of the gene expression between the young and mature thrombocytes revealed a high degree of similarity and less number of unique genes between these two thrombocyte populations. Our analysis also showed orthology between a large number of zebrafish thrombocyte and human megakaryocyte/platelet expressed genes. This information is valuable for the future knockdown studies of these genes to understand the mechanisms of thrombocyte and megakaryocyte maturation.

## Supporting information

S1 Raw images Amplification of a full-length of cDNA by PCR to generate sufficient mass for library construction. A 56 μL of cDNA amplification reaction was mixed to a 35 μL sample. 1,541 GFP+ mature thrombocytes (2^nd^ lane from left) and 2,176 RFP+ young thrombocytes (4^th^ lane from left) were loaded, and a 12 PCR cycle was performed. 1 μL of the sample (dilution factor 1:10) was run on Agilent 4200 Tapestation for cDNA QC and quantification. The total cDNA yield in ng is calculated by multiplying the cDNA concentration (pg/ μL) by the elution volume (40 μL) of post cDNA amplification reaction clean up the sample (taking any dilutions factors into account) and then divide by 1000 (pg/ng). The upper marker (1^st^ and 3^rd^ lanes from left) is labeled with a purple line, and the lower marker is labeled with a green line. The molecular weight products (from top 1500, 1000, 700, 500, 400, 300, 200, 100, 50, 25 bp) are indicated.
(PDF)

S1 File Extracted data from the raw values along with human orthologs and diseases associated with these genes corresponding to zebrafish transcript from GFP+ thrombocytes. (XLS)

S2 File Extracted data from the raw values along with human orthologs and diseases associated with these genes corresponding to zebrafish transcript from RFP+ thrombocytes. (XLS)

S3 File List of all genes as well as four categories of genes expressed in both GFP+ and RFP+ thrombocytes, used in heat map generation. (XLSX)

S4 File List of genes used in calculating the relative abundance of transcripts from both GFP+ and RFP+ thrombocytes. (XLSX)

S5 File List of transcripts from GFP+ thrombocytes used in TMP analysis. (XLSX)

S6 File List of transcripts from RFP+ thrombocytes used in TMP analysis. (XLSX)

S7 File List of transcripts from GFP+ thrombocytes used in logTMP analysis. (XLSX)

S8 File List of transcripts from RFP+ thrombocytes used in logTMP analysis. (XLSX)

S9 File z-score matrix values for genes expressed in GFP+ thrombocytes. (XLSX)

S10 File z-score matrix values for genes expressed in RFP+ thrombocytes. (XLSX)

S11 File List of highly expressed genes in GFP+ thrombocytes. (XLSX)

S12 File List of highly expressed genes in RFP+ thrombocytes. (XLSX)

S13 File List of expressed genes from both GFP+ and RFP+ thrombocytes used in quantile mapping. (XLSX)

S14 File GO enrichment analysis of expressed genes from GFP+ thrombocytes. (XLSX)

S15 File GO enrichment analysis of expressed genes from RFP+ thrombocytes. (XLSX)

S16 File Top 15 GO enrichment analysis of expressed genes from GFP+ and RFP+ thrombocytes. (XLSX)

S17 File KEGG analysis of expressed genes from GFP+ thrombocytes. (XLSX)

S18 File KEGG analysis of expressed genes from RFP+ thrombocytes. (XLSX)

S19 File List of highly expressed human megakaryocyte ortholog genes found in total thrombocytes. (XLSX)

S20 File List of not highly expressed human megakaryocyte ortholog genes found in total thrombocytes. (XLSX)

S21 File List of highly expressed human platelet transcripts found in total thrombocytes. (XLSX)

S22 File List of not highly expressed human platelet transcripts found in total thrombocytes. (XLSX)
