# Supplementary material for: Analysis of transcribed sequences from young and mature zebrafish thrombocytes
Source: PLoS One. 2022 Mar 23;17(3):e0264776. doi: 10.1371/journal.pone.0264776 (PMC8942222; doi:10.1371/journal.pone.0264776)
Supplement: S1 Raw images — A 56 μL of cDNA amplification reaction was mixed to a 35 μL sample. 1,541 GFP+ mature thrombocytes (2nd lane from left) and 2,176 RFP+ young thrombocytes (4th lane from left) were loaded, and a 12 PCR cycle was performed. 1 μL of the sample (dilution factor 1:10) was run on Agilent 4200 Tapestation for cDNA QC and quantification. The total cDNA yield in ng is calculated by multiplying the cDNA concentration (pg/ μL) by the elution volume (40 μL) of post cDNA amplification reaction clean up the sample (taking any dilutions factors into account) and then divide by 1000 (pg/ng). The upper marker (1st and 3rd lanes from left) is labeled with a purple line, and the lower marker is labeled with a green line. The molecular weight products (from top 1500, 1000, 700, 500, 400, 300, 200, 100, 50, 25 bp) are indicated. (PDF) [file pone.0264776.s001.pdf]

Lorem Ipsum

Lorem ipsum dolor sit amet, consectetur adipiscing elit. Mauris maximus fringilla ligula, in malesuada erat tempor ac. Quisque dapibus posuere turpis, vel aliquam massa vehicula non.

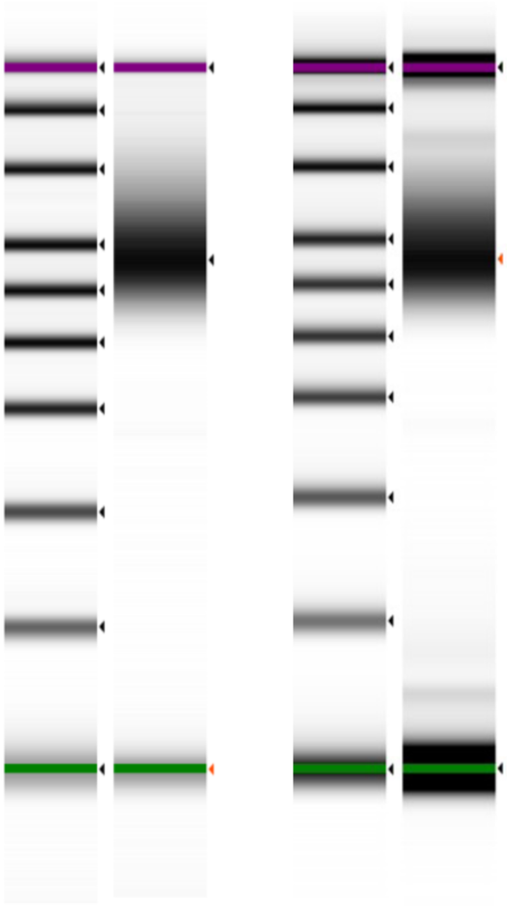

test-test-1 This is a preview of your figure rendered on a simulated PLOS journal page.

Maecenas ac est sit amet odio sollicitudin euismod. In risus odio, convallis a neque ac, varius ultricies arcu. Vestibulum et quam iaculis, ultricies odio et, molestie magna. Suspendisse vehicula purus id turpis eleifend, et convallis dui dignissim. Praesent tempus elit a metus sollicitudin, sed fringilla nulla porttitor. Nullam in tempus massa. Nunc maximus magna massa, nec volutpat risus rhoncus ut. Fusce quis ante sem. Aenean nulla nibh, tempus sit amet rhoncus at, eleifend vel risus. Sed dictum, sem ultrices elementum pharetra, lacus diam volutpat orci, scelerisque semper dui lacus ut enim.

Suspendisse in nunc id lacus commodo consequat. Proin semper aliquam varius. Fusce vitae neque aliquam nisi ultrices sodales vitae ut enim. Vivamus nec dictum ipsum. Sed condimentum ante eu urna tincidunt tincidunt. In ac lacus nec ipsum viverra volutpat posuere vel lacus. Class aptent taciti sociosqu ad litora torquent per conubia nostra, per inceptos himenaeos. Morbi rhoncus ipsum quis lorem hendrerit, at vulputate massa tempus. Ut arcu nisl, gravida vitae risus ultricies, porta venenatis massa. Cras dignissim, enim at faucibus aliquam, sapien nisl eleifend dolor, vel mollis nulla nisi id ipsum. Pellentesque vehicula ultricies risus sit amet faucibus. Praesent sit amet mi ac est faucibus accumsan. Praesent pulvinar sit amet orci auctor feugiat.
